# Gastric Parietal Cell and Intestinal Goblet Cell Secretion: a Novel Cell-Mediated In Vivo Metal Nanoparticle Metabolic Pathway Enhanced with Diarrhea Via Chinese Herbs

**DOI:** 10.1186/s11671-019-2908-z

**Published:** 2019-03-05

**Authors:** Yanlei Liu, Kunlu Liu, Meng Yang, Yue Han, Qian Zhang, João Conde, Yuming Yang, Gabriel Alfranca, Yuxia Wang, Lijun Ma, Yingge Zhang, Jie Song, Yunxiang Pan, Jian Ni, Daxiang Cui

**Affiliations:** 1 Institute of Nano Biomedicine and Engineering, Shanghai Engineering Research Center for Intelligent Instrument for Diagnosis and Therapy, Thin Film and Microfabrication Key Laboratory of Ministry of Education, Department of Instrument Science and Engineering, School of Electronic Information and Electronical Engineering, Shanghai Jiao Tong University, 800 Dongchuan Road, Shanghai, 200240 People’s Republic of China; 2 State Key Laboratory of Toxicology and Medical Countermeasures, Institute of Pharmacology and Toxicology, Academy of Military Medical Sciences, Beijing, 100850 People’s Republic of China; 3 School of Engineering and Materials Science, Queen Mary University of London, London, UK; 4 Department of Oncology, Tongren Hospital, Shanghai Jiao Tong University School of Medicine, 1111 Xianxia Road, Shanghai, 200336 People’s Republic of China; 5 National Center for Translational Medicine, Collaborative Innovational Center for System Biology, Shanghai Jiao Tong University, 800 Dongchuan Road, Shanghai, 200240 People’s Republic of China

**Keywords:** Triangular silver nanoplates, Magnetic nanoparticles, Au nanoclusters, Au nanorods, Goblet cells, Parietal cells intestinal excretion, CBD ligation

## Abstract

Up to date, the way in which metal nanoparticles are cleared in vivo has yet to be elucidated well. Herein, we report a novel intestinal goblet cell-mediated in vivo clearance pathway to remove metal nanoparticles. Typical metal nanoparticles such as triangular silver nanoplates, magnetic nanoparticles, gold nanorods, and gold nanoclusters were selected as representative examples. These metal nanoparticles were prepared, characterized, and injected via tail vein into a mice model with common bile duct (CBD) ligation. The feces and urines were collected for 7 days to be followed by the sacrifice of the mice and collection of the intestinal and gastric tissues for further analysis. The results showed that all four selected metal nanoparticles were located inside the goblet cells (GCs) of the whole intestinal tissue and were excreted into the gut lumen through the secretion of intestinal GC. Moreover, triangular silver nanoplates and gold nanorods were located inside the gastric parietal cells (PCs). Importantly, nanoparticles did not cause obvious pathological changes in intestinal tissues. In this study, we confirmed that the blood corpuscles are involved in the GCs secretion pathway. Furthermore, we found that the secretion of nanoparticles from intestinal GCs and PCs is accelerated by diarrhea induced via Chinese herbs. In conclusion, metal nanoparticles such as triangular silver nanoplates, magnetic nanoparticles, gold nanorods, and gold nanoclusters can be cleaned away by intestinal GCs and PCs. This novel pathway of in vivo clearance of metal nanoparticles has a great potential for future applications such as new drug design and development, nanoparticle-based labeling and in vivo tracking, and biosafety evaluation of in vivo nanoparticles.

**Electronic supplementary material:**

The online version of this article (10.1186/s11671-019-2908-z) contains supplementary material, which is available to authorized users.

## Introduction

With the rapid development of nanotechnology and its applications, a wide variety of engineered nanostructure materials are now used in pharmaceuticals, biomedical products, and other industries. The emerging nanotechnology products have an enormous potential for future economic growth and development, but the risks of nanotechnology on the environment and for human health are still not fully understood. To investigate the impact of nanoparticles on the human body, their interactions with biological systems, and their potential risk assessments, nanotoxicology has been looked as one novel multidisciplinary subject, drawing increasing attention of governments and scientists, and establishing the biosafety of nanomaterials as a key scientific problem. Up to date, many reports are closely associated with the interaction between nanoparticles and human cells. For instance, some nanoparticles such as graphene oxides, gold nanoclusters, and carbon dots can enter into the cytoplasm or cell nucleus, inducing cell cycle arrest or cell apoptosis, lung granuloma formation, and stimulating immunological cell secretion of some cytokines [1–3].

With the development of novel molecular imaging techniques, metal nanoparticles such as gold nanoparticles, silver nanoparticles, magnetic nanoparticles, and quantum dots have been actively investigated as multifunctional theranostic reagents, and are used for in vivo targeted imaging, magnetic-induced heating, photothermal or photodynamic therapy, or as high-efficient drug delivery systems, among other applications. It has been observed that these metal nanoparticle-based multifunctional nanoprobes are located in tumor sites, and part of them are also located in the liver and spleen tissues and can distribute throughout the kidney, lung, and brain tissues [4–10]. Because kidney only cleans away the nanoparticles with less than 5 nm in diameter, most nanoparticles are very difficult to be removed in this manner [11, 12]. Therefore, how to clean in vivo metal nanoparticles has become one challenging key scientific problem. However, to the present day, there are no convincing alternative pathways and detailed mechanisms to remove metal nanoparticles from the human body. Thus, how to clean metal nanoparticles in vivo has become our concern.

To date, metal nanoparticles introduced into the organism mainly by three routes, such as intravenous, oral, and intraperitoneal pathways, among which the intravenous injection is the most common method because of its rapid distribution throughout the entire body [4, 13, 14]. However, the degradation of the metal cores of these types of nanoparticles by the organism is, if possible, extremely difficult, leading to the primary problem, that is the effects of the accumulation of residual nanoparticles. It should be noted that the quality of in vivo metal nanoparticles is determined by the balance between nanoparticle-induced bioactivity and unwanted toxicity. From a toxicological perspective, a toxic effect is provoked only if sufficient amounts of nanoparticles are located in a target site, and the excretion from the organism is the best way to cease the effects of an excessive amount of nanoparticles located in the cells and tissues. Therefore, a proper understanding of their clearance pathways is crucial for any medical application and for a comprehensive risk assessment.

There are some studies associated with the clearance of nanoparticles from in vivo tissues or organs such as the kidney, liver, and lung [15–17]. However, these experiments merely provide information concerning the clearance mechanism to remove particles from the single organ instead of the whole body [18]. As to the systemic in vivo clearance, two main excretion pathways of intravenously injected nanoparticles have been reported, that is, the hepato-biliary system (HBS)-feces pathway for larger nanostructures that cannot be biodegraded by the organism like some types of magnetic nanoparticles [19, 20], and the kidney-urine pathway for small-sized nanoparticles, such as quantum dots, fullerenes, gold nanoclusters, and other types of gold nanoparticles with less than 5 nm in diameter [16, 21, 22]. However, these two pathways show limited clearance rate for in vivo metal nanoparticles.

Souris et al. demonstrated that silica nanoparticles accumulated in the intestinal wall at high concentration and that the concentration of intravenously injected 50–100 nm silica nanoparticles located in the liver was much lower than that in the intestinal wall and feces [20]. Another study showed that nanoparticles as large as 500 nm regardless of modifications can be eliminated from the fish body and that the elimination rate of 500 nm particles was faster and more efficient than that of 50 nm particles despite that the larger nanoparticles are far beyond the ability of HBS [23]. These data show that HBS pathway may not be the major excretion pathway of in vivo nanoparticles and that there may exist other excretion pathways for in vivo nanoparticles.

**Scheme 1:**
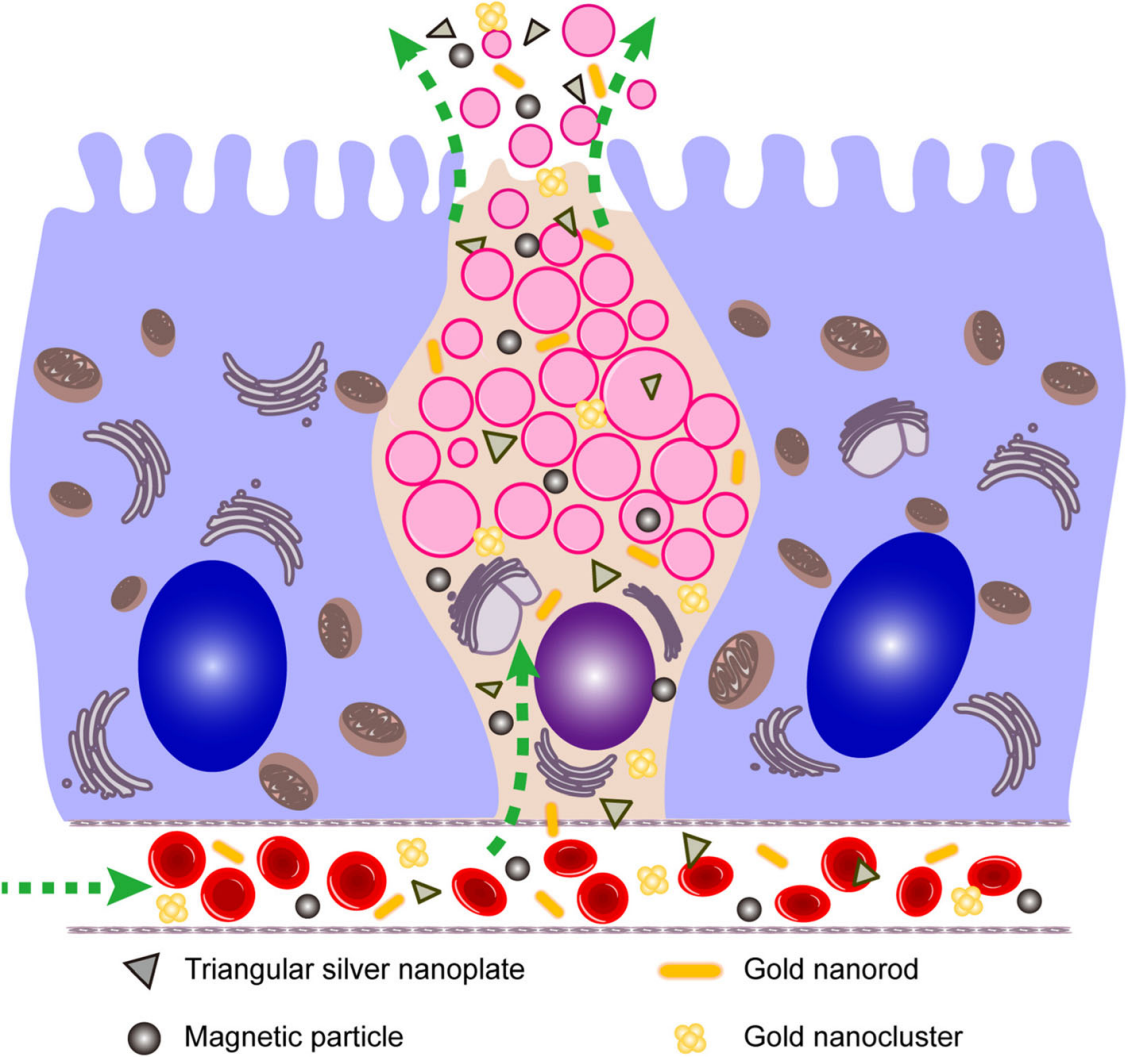
The intestinal GCs excretion pathway of nanoparticles

Intestinal goblet cells (GCs) are highly polarized excretory cells that are present throughout the intestinal tract. These specialized epithelial cells are thought to play an important protective role in the intestine by synthesizing and secreting several mediators [24–26]. Wang et al. reported that goblet cells (GCs) can uptake nanoparticles [27], and Sun et al. found that intravenously injected nanoparticles distributed in the intestinal GCs [28]. Nevertheless, up to date, the interaction between the nanoparticles and the intestinal GCs is still not investigated in detail. Specifically, no report fully clarifies how those nanoparticles are capable to get into the GCs cells, and whether those nanoparticles distribute in the GCs of the whole intestinal tissue. To clarify the excretion pathway of metal nanoparticles into the intestinal tissues, it is crucial to elucidate what role intestinal GCs play in this novel excretion pathway of nanoparticles. Because metal nanoparticles can be excreted through HBS and get into the gut, we have therefore focused on distinguishing between HBS-mediated excretion from that mediated by intestinal GCs (Scheme 1).

In this study, we selected four kinds of common metal nanoparticles such as magnetic nanoparticles, silver triangle nanoparticles, gold nanoclusters, and gold nanorods as research targets. Thanks to the characteristic optical properties of gold nanorods, they served as a tool to observe the intestinal distribution of nanoparticles by two-photon excitation, whereas the other three types of particles served as representative examples of various other metallic nanomaterials. Mice models were prepared with ligation of the common bile duct to prevent the connection between HBS and the intestinal tract. The metal nanoparticles were injected into mice via tail vein, then, nude mice were raised and feces were collected for 7 days, and the animals were finally sacrificed, and the intestinal tract tissues and gastric tissues were collected, prepared in slices, and finally analyzed using high-resolution transmission electroscope and ICP-MS to investigate the distribution of metal nanoparticles in the intestinal tissues. In addition, the presence of metal nanoparticles was measured in the feces of the mice with CBD ligation. Moreover, in this study, in order to further uncover the nanoparticle secretion mechanism of GCs and PCs, we used a recently developed mice diarrhea model induced via Chinese herbs.

## Materials and Methods

### Synthesis and Characterization of Triangular Silver Nanoplates

The triangular silver nanoplates were synthesized via procedures previously described by Mirkin [29] and colleagues with some modifications [30]. In a typical experiment at room temperature with air, AgNO_3_ (0.1 mM, 100 mL), trisodium citrate (30 mM, 6 mL), PVP (30 kDa molecular weight, 0.7 mM, 6 mL), and 240 μL H_2_O_2_ (30 wt%) were orderly added into a 250 mL flask. After vigorously shaking the combined solutions in the flask, 0.8 mL of a freshly prepared solution of 0.1 M NaBH_4_ was injected rapidly. Within a few seconds, the color of the solution turned yellow indicating the generation of silver nanospheres. In the next few hours, the flask was placed under sunlight or fluorescent lamp until the solution turned into blue color, without further color changes (at most 5 h). And the final solution was stored in 4 °C refrigerator for further use.

The absorbance spectrum of the prepared solution was measured by UV-vis-NIR spectrometer (UV-3600, Shimadzu, Japan) using a 1-cm optical path cuvette. The spectra were collected within a range from 200 to 950 nm with a 2 nm slit. The analysis by transmission electron microscopy was operated on JEM-200CX (JEOL, Japan) by dipping the carbon-coated copper TEM grid into the gathering nanoparticles in 1 mL deionized water after centrifuging a total of 10 mL of the solution in 1.5 mL microcentrifuge tubes at 6000 rpm for 30 min at 25 °C. A total number of 200 triangular silver nanoplates were selected from the TEM images to statistically compute the distribution of their edge sizes.

Superparamagnetic magnetite (Fe_3_O_4_) nanoparticles, gold nanoclusters, and gold nanorods were synthesized and characterized according to our previous reports [31–33] and stored at room temperature.

### Preparation of Animal Models with Ligation of Common Bile Duct

**Fig. 1:**
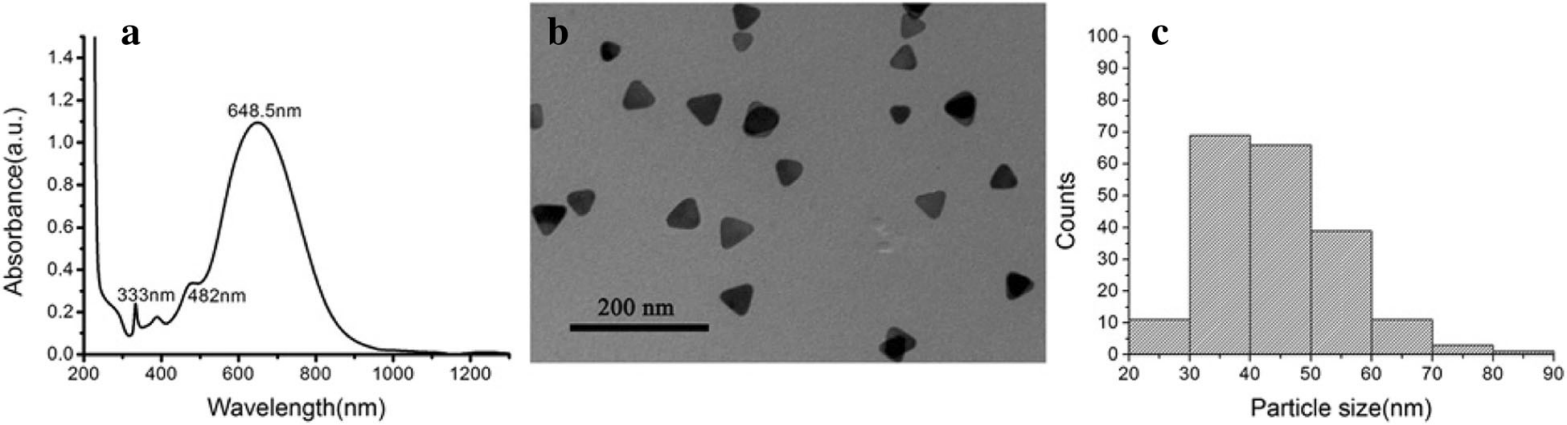
Characterization of the triangular silver nanoplates. **a** UV-vis spectrum of the prepared solution. **b** TEM image of the gathered silver nanoparticles after centrifugation. **c** Size distribution of the selected triangular silver nanoplates (200 nanoparticles from TEM image)

Healthy, female Wistar rats (180–220 g) and female rude mice (20–22 g) were obtained from Shanghai Slac Laboratory Animal Co. Ltd. (Shanghai, China). All animal experiments were performed in compliance with the relevant laws and institutional guidelines. All animal experiments were approved by the Institutional Animal Care and Use Committee of Shanghai Jiao Tong University (NO.SYXK2007-0025). The common bile duct was ligated following a method originally described by Lee with some modifications [34]. Briefly, these mice were anesthetized with pentobarbital (25 mg/kg) and fixed onto a wooden surgical sheet. A mid-abdominal incision was made, and the abdominal tissues were separated carefully to clearly expose the CBD. Two sterile nylon medical surgical sutures (Shanghai Jinhuan Industry CO., Ltd., Shanghai, China), 0.2 mm in diameter, were put through under the CBD, and three nodes were made at both ends of a segment of the CBD (Fig. 1b, c). Finally, the CBD was then cut off between the two ends, followed by the final closure of the abdomen. On the 14th day after ligation of the common bile duct, blood samples were collected from each mouse to test the major hepatic function.

### Injection of Nanoparticles into the Mice

After finishing the CBD ligation, 12 mice were randomly divided into four groups: control group 1, silver nanoplates test group, magnetic nanoparticles test group, and gold nanoclusters test group. An additional control group was comprised of five mice without CBD ligation. The mice in control groups were treated with an intravenous injection of 0.9% NaCl aqueous solution, while the test groups were injected with a nanoparticle suspension such as triangular silver nanoplates, magnetic nanoparticles, gold nanorods, and gold nanoclusters at a dose of 150 μL (550 μg/mL). All four suspensions of nanoparticles were freshly dispersed by sonication for 1 min before use. The mice were anesthetized by inhalation of 5% isoflurane until muscular tonus relax, then four kinds of nanoparticle suspensions were intravenously injected using 1 mL syringe, respectively.

### Distribution of Nanoparticles in the Tissues

On the seventh day after injection of the metal nanoparticle suspension, the mice were anesthetized and their intestinal tissues were taken out, fixed in 10% formaldehyde for 24 h and then paraffin-imbedded. A Leica RM2135 Rotary Microtome was used to prepare 5-μm-thick sections of the fixed samples. Finally, the sections were dehydrated with alcohol and stained in hematoxylin and eosin. The sections of the samples were observed under a phase contrast microscope (Olympus, RX-71, Japan).

On the seventh day after injection of the metal nanoparticle suspension, the intestinal tissues and gastric tissues were collected immediately after scarifying the mice and were fixed in 2.5% glutaraldehyde solution. The fixed samples were dehydrogenated serially in ethanol and embedded with epoxy resin. After that, ultrathin intestinal specimen was made and observed with high-resolution TEM (FEI, Tecnai G2 Spirit Biotwin, USA).

On that same day, their intestinal tissues were collected immediately after the sacrifice and were imaged by using an in vivo imaging system (IVIS-100 imaging system, Caliper) coupled with a cool charge-coupled device (CCD) camera and a red fluorescent protein (DsRed) filter (Caliper Life Sciences). Images and measurements of fluorescent signals were acquired and analyzed by Living Image 3.2 software (Caliper Life Sciences).

### Metal Content of the Feces

In addition, all of the mice feces were collected within 7 days after the injection, and the feces were weighed and digested with aqua regia under heating. Finally, the metal mental content in the solution was determined by an ICP-MS (Agilent 7500a, USA).

### Preparation of Chinese Herbal Extracts

Senna leaf 10 g, rhubarb 2 g, and fructus cannabis extracts 1 g were added to 100 mL water, heated to 100 °C for 10 min, and then filtered by two layers of gauze [35]. Finally, the filtrates were collected and concentrated to 0.3 g/mL under reduced pressure. The extracts of senna leaf were prepared as below and stored at 4 °C before the tests were performed.

### Goblet Cell Analysis

Firstly, six male Kunming mice were randomly separated into two groups: the control group and the diarrhea group; both were treated with saline and Chinese herbal extracts daily for 7 days via oral gavage (0.1 mL), respectively. The seventh day after gavage administration, the mice were sacrificed, the intestinal tissues were collected, and the intestinal and gastric tissues were frozen in Tissue Tek OCT and sectioned on a Leica CM 1510 S cryostat (Sakura Funetek, USA). The 8 μm sections were stained in Alcian Blue (1% Alcain Blue 8GX in 3% glacial acetic acid) for 5 min, and were finally rinsed in distilled water. This sample was oxidized in 1% periodic acid before washing and then treated for 15 min in Schiff’s reagent. Images of the tissue sections were recorded using an inverted microscope. The gastric tissues were collected on positively charged slides for two-photon luminescence imaging.

### Gold Content of the Intestinal Tissues and Feces

Briefly, 9 male Kunming mice were divided into each of the three groups according to the different treatment: control group, ligation groups, and ligation + diarrhea groups. Then, these mice were intravenously injected with 100 μL GNRs (1 mg/mL). The second day after tail vein injection, the control and ligation groups were treated with saline, at the same time the ligation + diarrhea groups were treated with Chinese herbal extracts. The dose of the treatment was kept constant and delivered daily for the following 7 days via oral gavage (0.1 mL). On the seventh day, the mice were sacrificed, and the intestinal tissues were frozen in Tissue Tek OCT and sectioned on a Leica CM 1510 S cryostat (Sakura Funetek, USA). Sections (8 μm) were collected on positively charged slides for two-photon luminescence imaging. All of the mice feces were collected after injection. The feces were weighed and digested with aqua regia under heating. Finally, the gold mental content in the solution was determined by an ICP-MS (Agilent 7500a, USA).

### Statistical Analysis

Each experiment was repeated three times in duplicate. The results were presented as mean ± SD. Statistical differences were evaluated using the *t* test and considered at *P* < 0.05.

## Results and Discussion

### Synthesis and Characterization of the Nanoparticles

Triangular silver nanoplates were synthesized by rapid thermal synthesis method, exhibiting good water solubility. More importantly, the particular triangular shape of these nanoparticles makes it easy to be identified by electron microscopy. As shown in Fig. 1, in the UV-vis spectrum, prepared silver nanoparticles showed a strong peak at 648.5 nm corresponding to the in-plane dipole surface plasmon band and two modest peaks at lower wavelengths, corresponding to the in-plane (482 nm) and out-of-plane (333 nm) quadrupole resonances, indicating the formation of triangle architecture [36], which is further verified by the TEM image of the silver nanoparticles gathered after centrifugation. TEM image (Fig. 1b) revealed that the prepared batches did contain a subpopulation of silver nanospheres, possibly contributing to the SPR peak at 389 nm [36]. The edge length of the gathered triangular silver nanoplates was 44.3 nm with good monodispersed distribution.

Magnetic nanoparticles with 20 nm in diameter and Au nanoclusters with 5 nm in diameter were prepared, and their characterization is shown in Additional file 1: Figure S1 and S2 respectively. The TEM images and UV/vis spectra of Au nanorods are shown in Additional file 1: Figure S3.

### Preparation of the CBD Ligation Mice Models

**Fig. 2:**
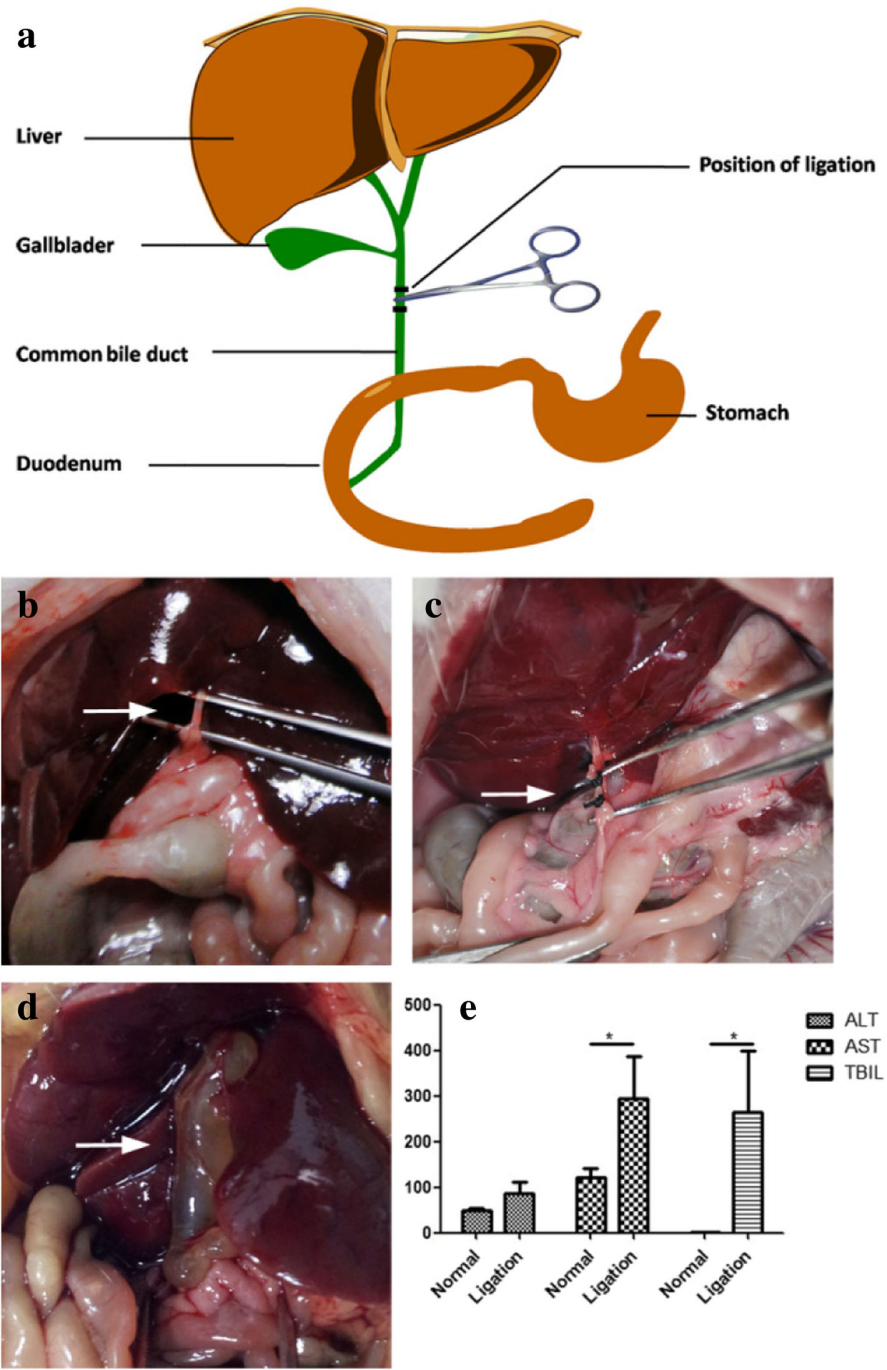
**a** A schematic illustration of the relations of HBS with intestinal tract. **b, c** Ligation of CBD (white arrows). **d** CBD swelling on the 14th day after CBD ligation (white arrow). **e** Examination of the major liver function. *P < 0.05

Common bile duct (CBD) ligation is a well-known experimental model used to induce liver cholestatic fibrosis [37, 38]. Here, we carried out experiments in mice with CBD ligation in order to totally block the connection between HBS and intestinal tract (Fig. 2a, b), making sure that metal nanoplates were transported only by the bloodstream to the intestinal tissues after intravenous injection. Compared with normal controls, the treated groups showed a strong increase in the diameter and thickness of the common bile duct wall 14 days after CBD ligation due to bile stasis (Fig. 2d). In addition, as shown in Fig. 2e, the levels of TBIL and AST in the ligation group were significantly higher than in the contrast group. These results suggested that after successfully building the CBD ligation mice model, the common bile duct was absolutely blocked and that the connection between HBS and the intestinal tract was completely cut off, thus inducing cholestasis and liver cholestatic fibrosis [39].

### The Effect of Four Kinds of Nanoparticles on the Intestinal Tissues

**Fig. 3:**
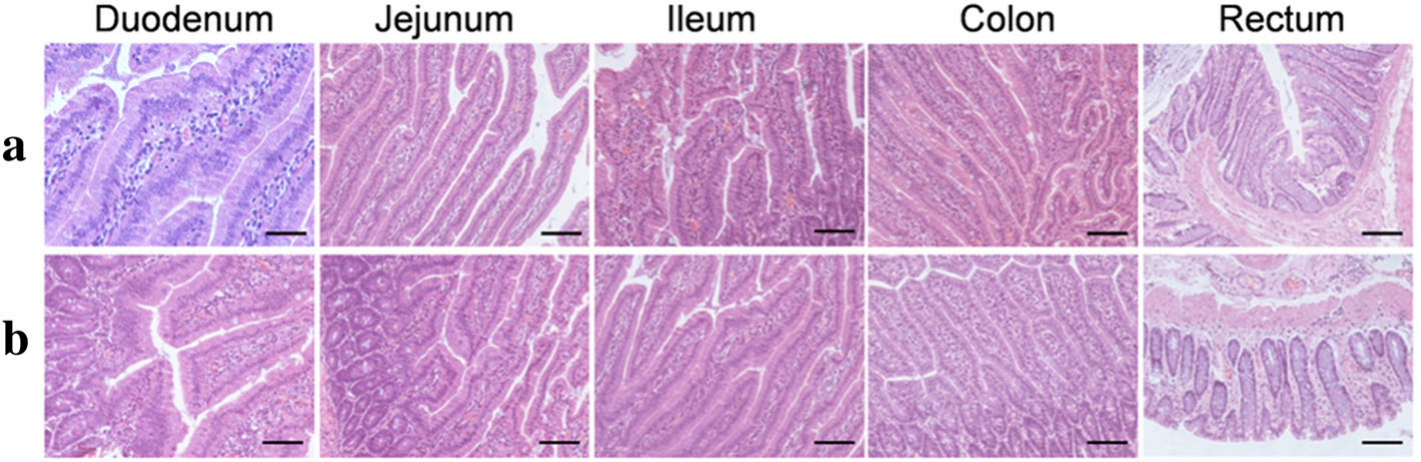
Histopathological microsection of different intestinal tissue samples of mice with CBD ligation. **a** Control groups: mice treated with saline injection via tail veins (upper panels). **b** Test groups: mice treated with triangular silver nanoplates suspension injected via tail veins (lower panels)

Normally, the intestinal epithelium provides a semi-permeable barrier which allows small amounts of molecules of different sizes and characteristics to cross the intact epithelium by both active and passive mechanisms. Generally, the larger the molecule, the less likely is for it to cross this barrier. However, once the gut lining gets inflamed or damaged, it becomes more difficult for the intestinal epithelium to keep foreign and large particles out as the spaces between cells open up [40, 41]. Considering that nanoparticles may be the cause of the pathological changes of intestinal tissues and the consequent increase of permeability of the intestinal wall, which leads the nanoparticles to pass the intestinal wall through, we carried out a histopathological examination of the intestinal tissues after being exposed to four different types of nanoparticles: magnetic nanoparticles, silver triangle nanoparticles, gold nanorods, and gold nanoclusters. As shown in Fig. 3, no significant differences were observed between control groups and test groups, nor were there other histological changes such as inflammatory infiltrate [42]. The results demonstrate that these metal nanoparticles caused no pathological change of intestinal tissues, thus eliminating the possibility that nanoparticles leak from the spaces between cells.

### Distribution of Metal Nanoparticles in Intestinal GCs

**Fig. 4:**
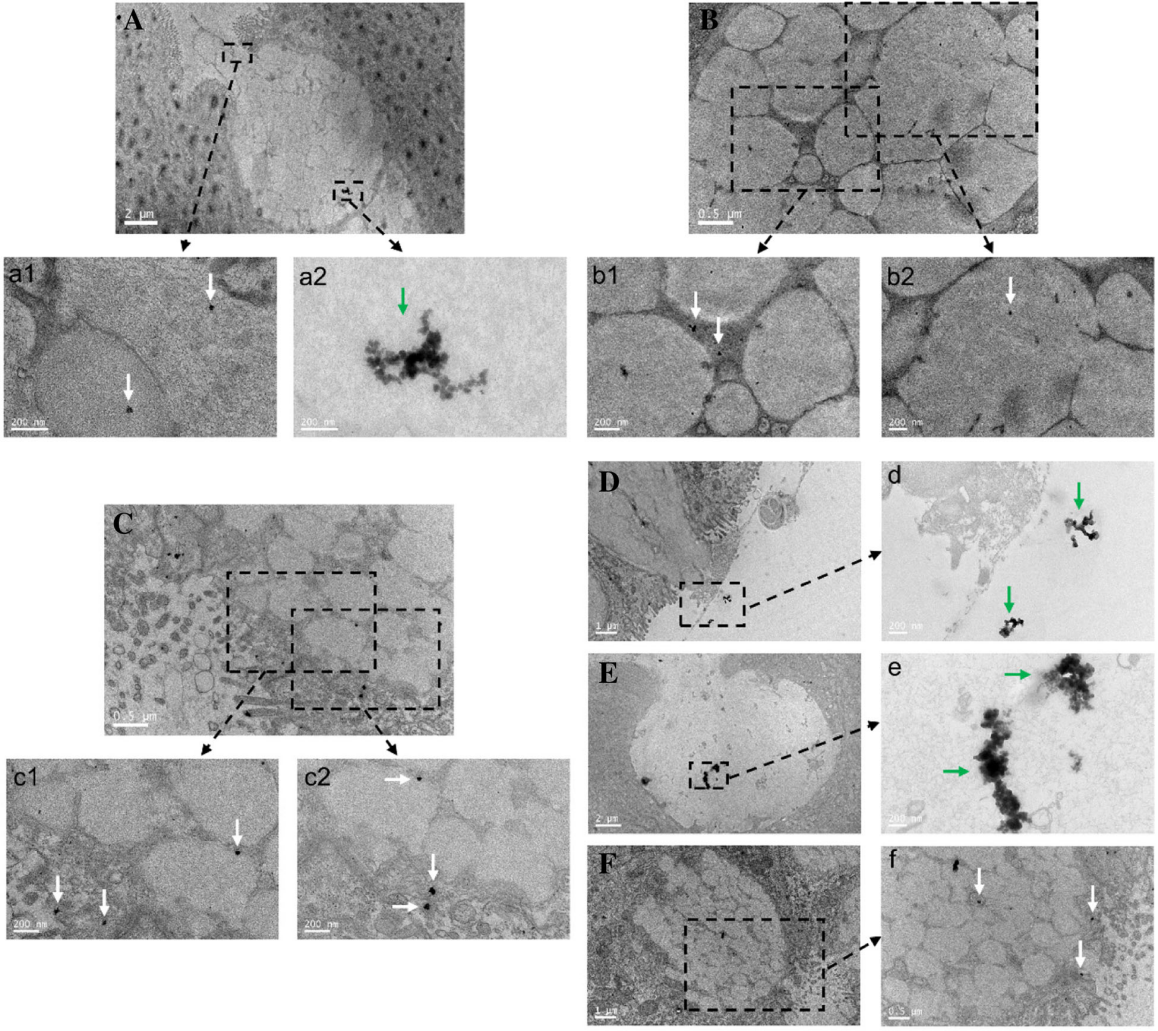
Distribution of triangular silver nanoplates in intestinal GCs of mice with CBD ligation. The CBD ligation mice group was treated with triangular silver nanoplates injected via tail vein 7 days after ligation. Intestinal GCs of different intestinal tissues. **A** Duodenum, triangular silver nanoplates were shown in an aggregation mode (green arrow), while some triangular sliver nanoplates were in a dispersion mode (white arrows). **B** Jejunum, triangular silver nanoplates located at the intestinal GC (white arrows). **C** Ileum and some triangular silver nanoplates were excreted out, while some were still inside. **D** Colon, some triangular silver nanoplates were secreted out and into the gut. **E**, **F** Rectum, some triangular silver nanoplates were ready to excrete out (dispersion mode, white arrows), while others were still inside (aggregation mode, white arrow)

GCs are one type of the four main cell types present throughout the intestinal tract and are responsible for the production and preservation of a protective mucus blanket by synthesizing and secreting high-molecular-weight glycoproteins known as mucins, which promote the elimination of gut contents and provide the first line of defense against physical and chemical injury caused by ingested food, microbes, and the microbial product [43, 44]. The GCs were easily identified thanks to their high volume of mucus content. As shown in Fig. 6, the triangular silver nanoplates were located inside the intestinal GCs throughout the intestinal tract, and the different phases of their secretion from GCs could be obtained. Figure 4d shows how some triangular silver nanoplates had been secreted out and into the gut by a GC. Figure 4e shows that some triangular silver nanoplates encapsulated in the mucus contents of the intestinal GCs were ready to be secreted. In Fig. 4f, it is displayed how some triangular silver nanoplates had been expelled out from a GC, while others were still in it. From the TEM images, we found that some triangular silver nanoplates were shown in an aggregation mode (Fig. 4 (a2, d and e), green arrows), while others were in a dispersion mode (Fig. 4 (a1, b1, b2, c1, c2 and f), white arrows). Aggregation is a common phenomenon of nanoparticles, and it is generally observed when their concentration is greatly increased in cells [45]. On the contrary, a decrease in the concentration of nanoparticles prevents their aggregation.

Similar results were observed for gold nanoclusters, magnetic nanoparticles, and gold nanorods, as shown in Additional file 1: Figure S4, S5, and S6. These results clearly showed that these three kinds of metal nanoparticles are located inside GCs of the intestinal tract, indirectly supporting that these metal nanoparticles may be cleaned away by the GCs pathway.

Although GCs are distributed along the entire line of the intestinal tract, their contribution to the total epithelial volume is not identical. In the small intestine of mice, the volume density of GCs increases progressively from the duodenum to ileum. This trend continues in the large intestinal tract with the density of GCs in the colonic epithelium also increasing proximal to distal, from the colon to rectum [43]. Based on the fact that triangular silver nanoplates, magnetic nanoparticles, and gold nanoclusters existed in the intestinal GCs throughout the intestinal tract, and that the contribution of GCs to the total epithelial volume is totally different, we believe that the large intestine may be the main excretion place for the intestinal GCs excretion pathway.

Due to their characteristic shape, triangular silver nanoplates were easily distinguished by TEM imaging in the locations described in the suggested pathway to reach the GCs. However, although magnetic nanoparticles and gold nanoclusters were not able to be distinguished from other structures using this technique, Additional file 1: Figure S4 reveals that the intestinal tract of the CBD ligation group still has a certain amount of gold nanoclusters which leads us to the conclusion that the above-mentioned mechanism of GC excretion is also applied for other types of metal nanoparticles.

In addition, as Additional file 1: Figure S7 shown, the ICP-MS results clearly exhibits that these nanoparticles can still be secreted from the ligation mice body. These results proved that the goblet cells of intestinal tissues are involved in an important pathway for the excretion of nanoparticles.

### Potential Mechanism of Transport of Metal Nanoparticles in the Intestinal Blood Vessel

**Fig. 5:**
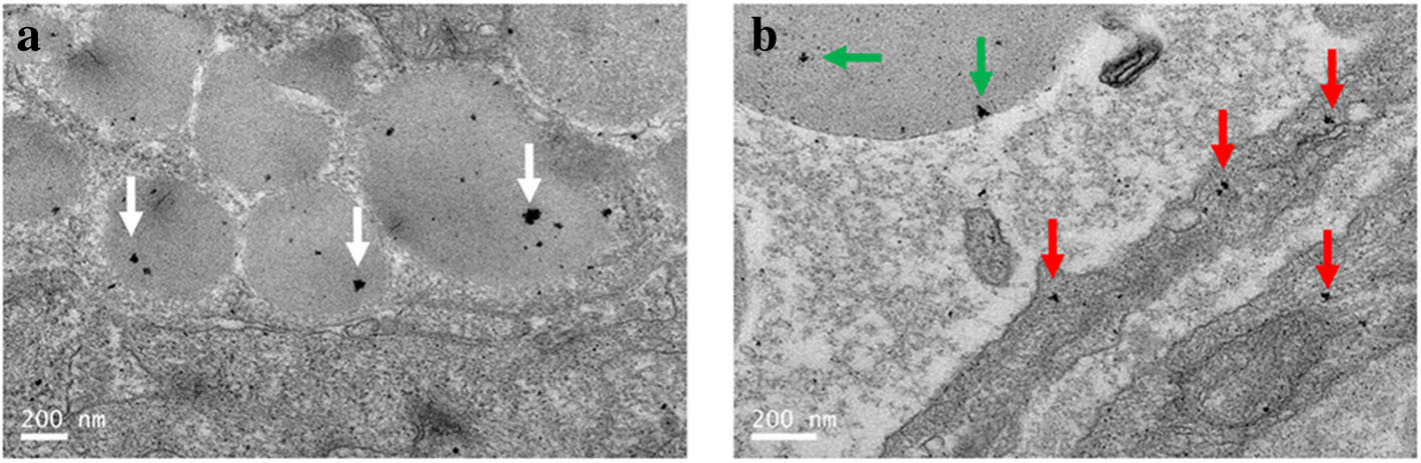
Distribution of triangular silver nanoplates in the intestinal vessels of mice with CBD ligation. **a** Triangular silver nanoplates located in the blood corpuscle (white arrows). **b** Triangular silver nanoplates penetrated into the vascular wall (green arrows), while some located in the blood corpuscle (red arrows)

The results mentioned above demonstrate that these four kinds of metal nanoparticles (magnetic nanoparticles, silver triangle nanoparticles, gold nanorods, and gold nanoclusters) were distributed in the GCs throughout the intestinal tract, but the way in which the nanoparticles enter the GCs was still not elucidated. Because the mouse models with CBD ligation were treated with a suspension of metal nanoparticles via tail vein injection, these nanoparticles could only be transported by the blood flow into the intestinal vessels. As revealed by TEM imaging, some triangular silver nanoplates were indeed located in the blood corpuscle (Fig. 5a, white arrows). What is more, previous studies have revealed that nanoparticles with a small size can be delivered by the blood corpuscle all over the circulatory system [46]. It can be seen in Fig. 5b that some triangular silver nanoplates pass through the membrane of the blood vessels (green arrows), while some triangular silver nanoplates were located in the blood corpuscle (red arrows). Therefore, as Fig. 8 reveals, we infer that triangular silver nanoplates were transported by the blood corpuscles and then were released into the plasma, followed by passing the membrane of the vascular wall of the intestinal vessels and finally arriving at the GCs.

### Goblet Cell Analysis Assay

**Fig. 6:**
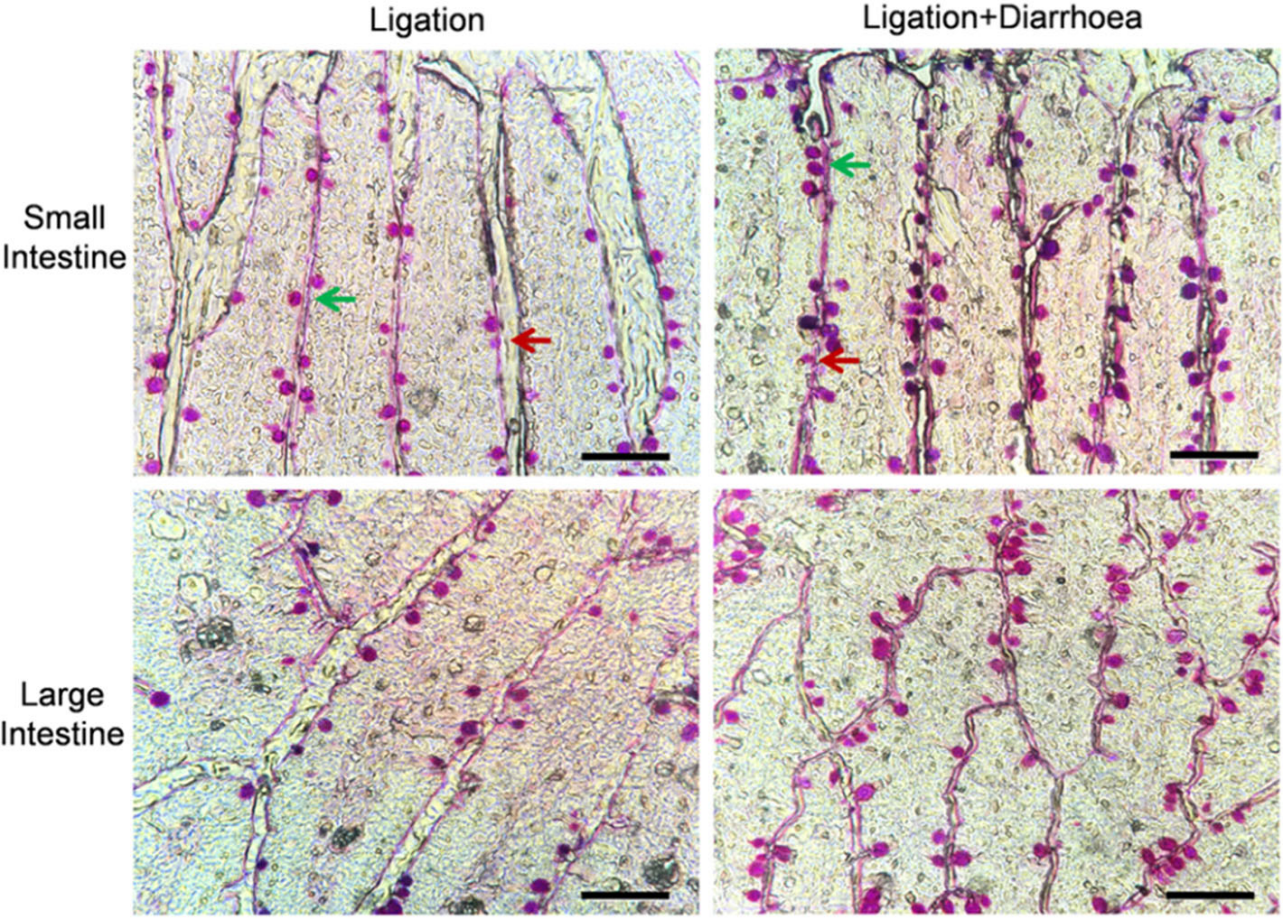
Photomicrographs of intestinal tissue stained with Alcian Blue/Schiff’s reagent to visualize goblet cells. Images are representative of mice treated with saline (Ligation groups) and senna leaf (ligation + diarrhea groups) with arrows indicating non-cavitated goblet cell (green arrow) and cavitated goblet cell (red arrow) secreting mucin. All bars are 100 μm

Goblet cells play a key role in the excretion pathway of nanoparticles. In this study, we found that metal nanoparticles can be secreted from these goblet cells. Following this statement, if the secretion process of the goblet cells is accelerated, it would theoretically be possible that the excretion of nanoparticles will also increase. To address this, we established a diarrhea model induced by a Chinese herb used in traditional medicine. In order to explore how diarrheic processes influence the secretion of GCs, a histological analysis of the intestinal GCs was conducted. It must be acknowledged that an increased number of intestinal tissue goblet cells increases the mucin production [47]. As shown in Fig. 6, in the diarrhea groups, the total number of goblet cells in the small intestinal and the large intestinal were significantly higher compared to the controls. In addition, the percentage and number of cavitated goblet cells in the intestinal tissues were significantly higher in the diarrhea groups compared to the controls. These observations let us assert that the amount of intestinal tissue cells increases in response to diarrhea, suggesting an increased excretion by the GCs. These results are consistent with data reported previously [47].

### Gold Contents of the Intestinal Tissues and the Feces

**Fig. 7:**
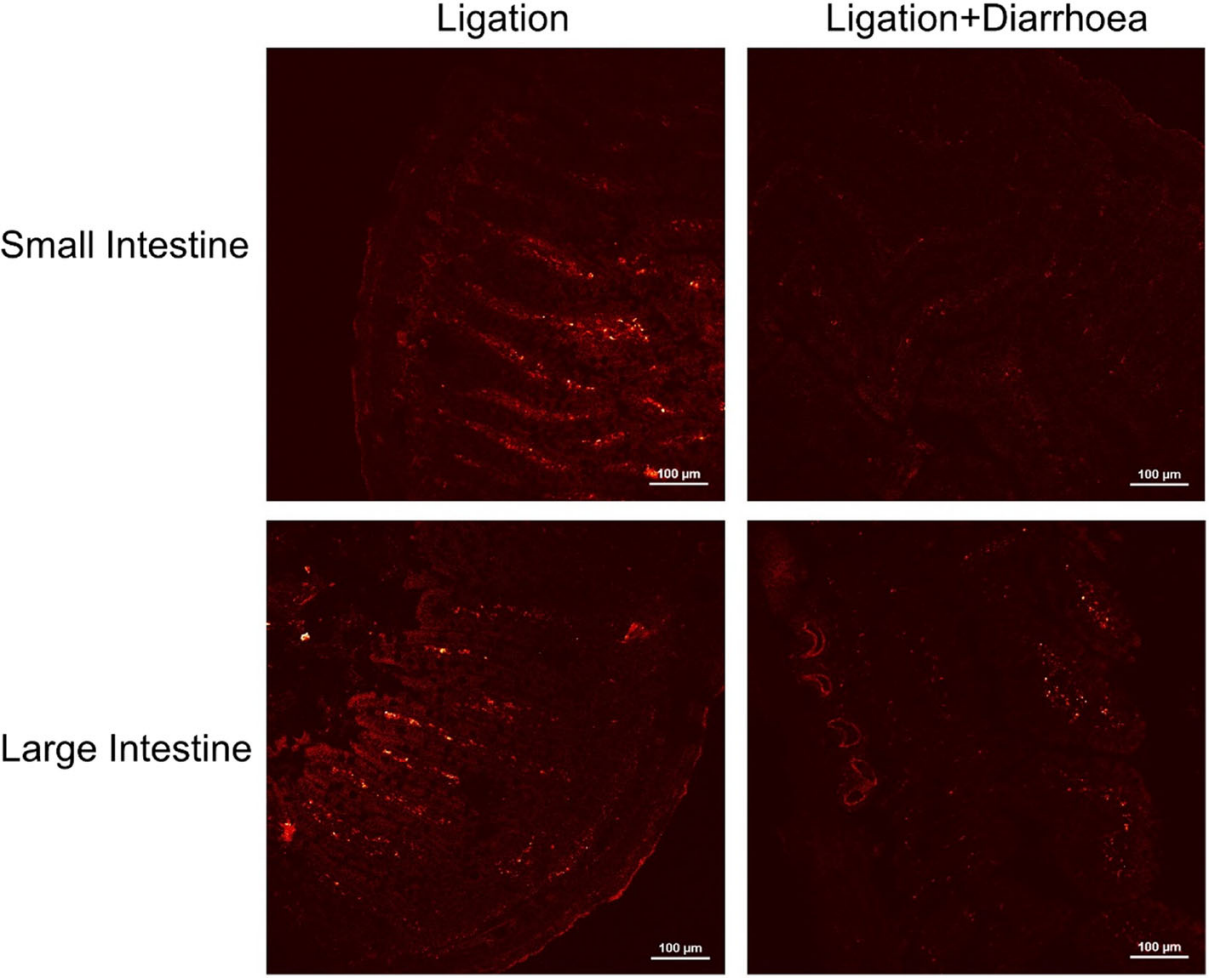
Two-photon-laser scanning confocal microscopy images of intestinal tissue sections at 7 days after tail vein injection of GNRs (excitation 780 nm, emission 601–657 nm)

It has been reported that the two-photon action cross-section (TPACS) of a nanorod can reach 2320 GM, which is much higher than that of organic fluorophores and within the range of that of quantum dots, providing a promising approach to detect the distribution of the gold nanorods in biological tissues using two-photon excitation [48, 49]. In this part of the study, in order to observe the intestinal distribution of nanoparticles in the ligation groups and the ligation + diarrhea groups, two-photon luminescence of the AuNRs core was measured. As shown in Fig. 7, the gold contents of small and large intestinal were significantly higher for the ligation groups compared with those observed in ligation + diarrhea groups. The gold elemental contents in intestinal tissues were quantified by ICP-MS 7 days after tail vein injection. The gold contents of intestinal tissues were significantly higher in the ligation groups compared to that in the ligation + diarrhea groups (*P* < 0.001) throughout the study (Fig. 7). These results indicate that the level of excreted nanoparticles by the goblet cells of the diarrhea groups is higher than that of any of the other groups.

**Fig. 8:**
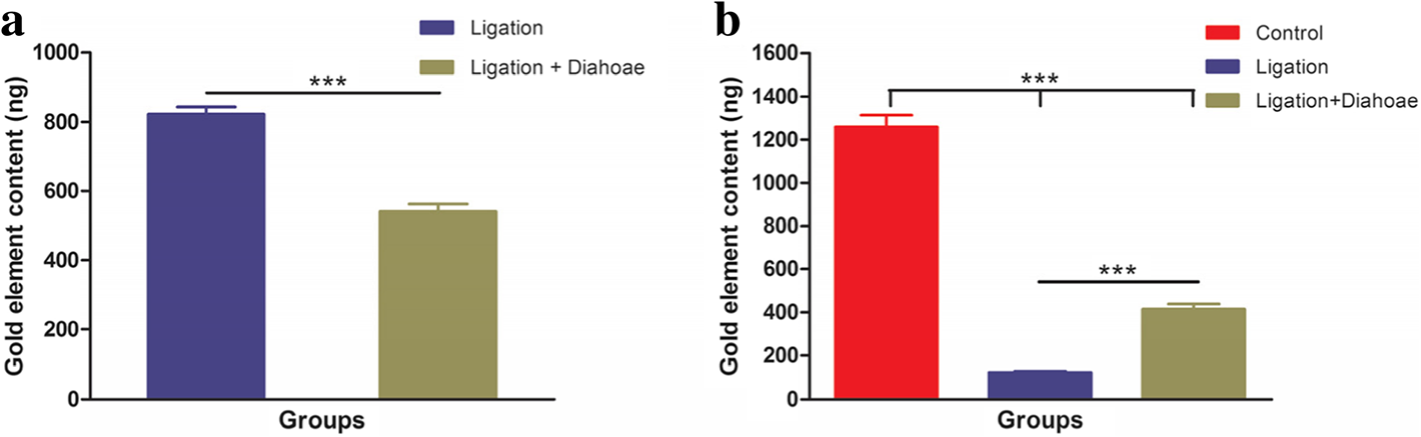
Content of GNRs at the intestinal tissues 7 days after injection (**a**), and gold element content of feces (**b**) based on ICP-MS analysis. ***P < 0.01, showing a significant difference between ligation groups and ligation + diarrhea groups

In the next experiment, we analyzed the gold contents in the feces of mice. As shown in Fig. 8a, the gold contents of feces were significantly higher in the control group compared to that in the ligation groups or the ligation + diarrhea groups (*P* < 0.001). Moreover, in the ligation + diarrhea groups, the gold contents were significantly higher than in the ligation groups (Fig. 8a). These results suggest that diarrhea accelerates the process of nanoparticles being secreted by the intestinal goblets cells. Combined with quantitative analysis of gold elements in feces, we further proved that the goblet cells of intestinal tissues are involved in an important pathway for the excretion of nanoparticles.

### Effects of Parietal Cells on Gastric Secretion of Metal Nanoparticles

**Fig. 9:**
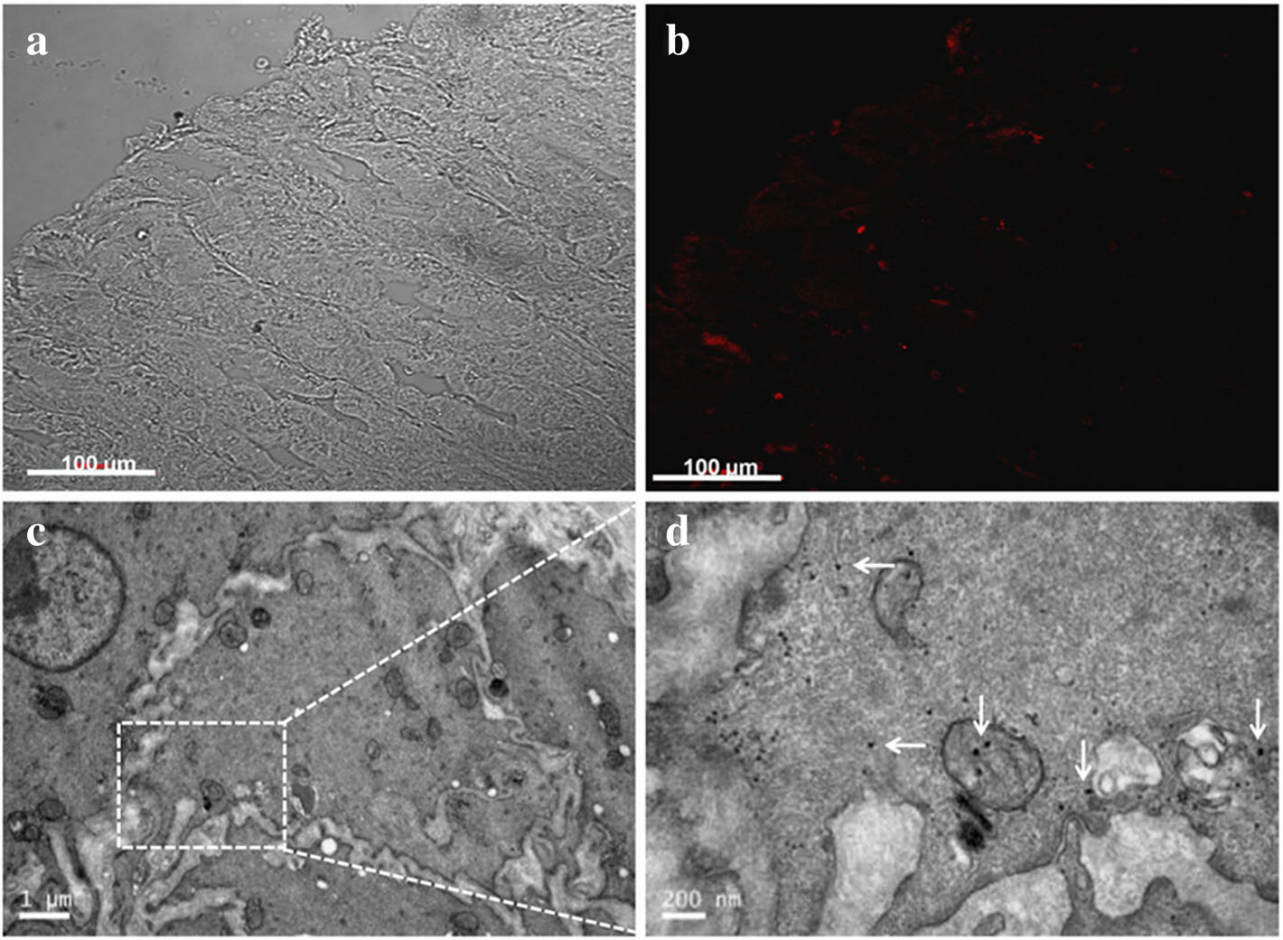
Distribution of nanoparticles in the gastric tissue. (**a**) and (**b**): Two-photo-laser scanning confocal microscopy images of intestinal tissue sections 7 days after tail vein injection of GNRs (Excitation: 780 nm, Emission: 601-657 nm). (**c**) TEM image of the gastric parietal cells; (**d**) Triangular silver nanoplates located in the gastric parietal cells (white arrows)

Parietal cells are mainly distributed in the bottom of the stomach and the gastric body, which secrete hydrochloric acid and internal factor. Furthermore, we found that gold nanoclusters distributed in the gastric tissues of mice with CBD ligation (Additional file 1: Figure S4B). Therefore, we hypothesized that parietal cells may be involved in the excretion of nanoparticles. As expected, from two-photon luminescence images, it was found that gold nanorods are distributed in the gastric tissues (Fig. 9a, b). In addition, as Fig. 9c, d shows, we observed that triangular silver nanoplates are distributed in the parietal cells of gastric tissue. Combined with the previous research results, we speculate that the parietal cells are involved in the secretion of nanoparticles.

## Conclusions

In summary, we successfully prepared and applied triangular silver nanoplates, magnetic nanoparticles, gold nanorods, and gold nanoclusters as tracking agents. Mice with CBD ligation were treated with the previously prepared nanoparticles via tail vein injection to study the gastric-intestinal tissue distribution and excretion of these nanoparticles. We also analyzed the excretion pathways of gold nanoclusters and magnetic nanoparticles. It must be stated that gold nanoclusters are mainly cleaned away via kidney urinary pathway, whereas magnetic nanoparticles are mainly removed from the organism via HBS pathway. As the excretory capabilities of kidney and HBS for in vivo applications of metal nanoparticles are very limited, the GCs and PCs excretion pathway may provide another important alternative way for the excretion of these nanoparticles. Concerning this issue, we also found that the process of nanoparticles secreted from GCs and PCs is accelerated by diarrhea, further proving that the GCs and PCs represent an important pathway for the excretion of metal nanoparticles. Admittedly, our knowledge is still limited with respect to the in vivo clearance of nanoparticles as, for example, the concrete mechanism underlying the GCs and PCs secretion pathways, and the clearance efficiency of nanoparticles in intestinal GCs, thus further investigations are urgently needed. To sum up, this novel pathway of in vivo clearance of metal nanoparticles has great potential in short-term applications such as new drug design and development, nanoparticle-based labeling and in vivo tracking, and biosafety evaluation of in vivo nanoparticles.

## Additional file

Additional file 1: **Figure S1.** Characterization of Fe_3_O_4_ magnetic nanoparticles by HR-TEM. Figure S2. Characterization of Au clusters by HR-TEM. Scale bar, 5 nm. Figure S3. (A) TEM image of GNRs. Scale bar, 100 nm; (B) absorption spectra of GNRs. Figure S4. Distribution of Au nanoclusters in intestinal tissues. (A) Control groups; (B) ligation groups. Figure S5. Distribution of magnetic nanoparticles in goblet cells of mice with CBD ligation. Figure S6. Distribution of gold nanorods in goblet cells of mice with CBD ligation. Figure S7. Quantitative analysis of nanoparticles in feces based on ICP-MS. (DOCX 2987 kb)

## Abbreviations

CBD: Common bile duct
CCD: Charge-coupled device
GCs: Goblet cells
GNRs: Gold nanorods
HBS: Hepato-biliary
PCs: Parietal cells
System Fe_3_O_4_: Superparamagnetic magnetite
TEM: Transmission electron microscopy
TPACS: Two-photon action cross-section
