# Supplementary material for: Gastric Parietal Cell and Intestinal Goblet Cell Secretion: a Novel Cell-Mediated In Vivo Metal Nanoparticle Metabolic Pathway Enhanced with Diarrhea Via Chinese Herbs
Source: Nanoscale Res Lett. 2019 Mar 5;14:79. doi: 10.1186/s11671-019-2908-z (PMC6401067; doi:10.1186/s11671-019-2908-z)
Supplement: Supplementary file 1 — Figure S1. Characterization of Fe3O4 magnetic nanoparticles by HR-TEM. Figure S2. Characterization of Au clusters by HR-TEM. Scale bar, 5 nm. Figure S3. (A) TEM image of GNRs. Scale bar, 100 nm; (B) absorption spectra of GNRs. Figure S4. Distribution of Au nanoclusters in intestinal tissues. (A) Control groups; (B) ligation groups. Figure S5. Distribution of magnetic nanoparticles in goblet cells of mice with CBD ligation. Figure S6. Distribution of gold nanorods in goblet cells of mice with CBD ligation. Figure S7. Quantitative analysis of nanoparticles in feces based on ICP-MS. (DOCX 2987 kb) [file 11671_2019_2908_MOESM1_ESM.docx]

Supporting information

**Gastric parietal cell and Intestinal goblet cell secretion: a novel cell-mediated *in vivo* metal nanoparticle metabolic pathway enhanced with diarrhea via Chinese Herbs**

Yanlei Liu^1^, Kunlu Liu^2^, Meng Yang^1^, Yue Han^1^, Qian Zhang^1^, João Conde^3^, Yuming Yang^1^, Gabriel Alfranca^1^, Yuxia Wang^2^ , Lijun Ma^4,5^, Yingge Zhang^2^, Jie Song^1^, Yunxiang Pan^1^, Jian Ni^1,5^, Daxiang Cui^1,5^


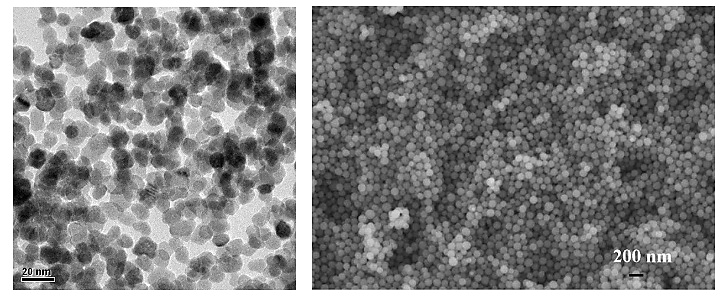
**Figure S1.**Characterization of Fe_3_O_4_ magnetic nanoparticles by HR-TEM.


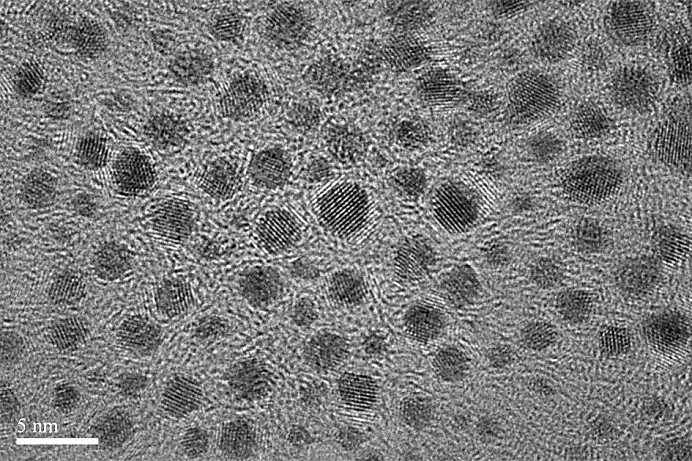


**Figure S2.** Characterization of Au clusters by HR-TEM. Scale bar, 5 nm.


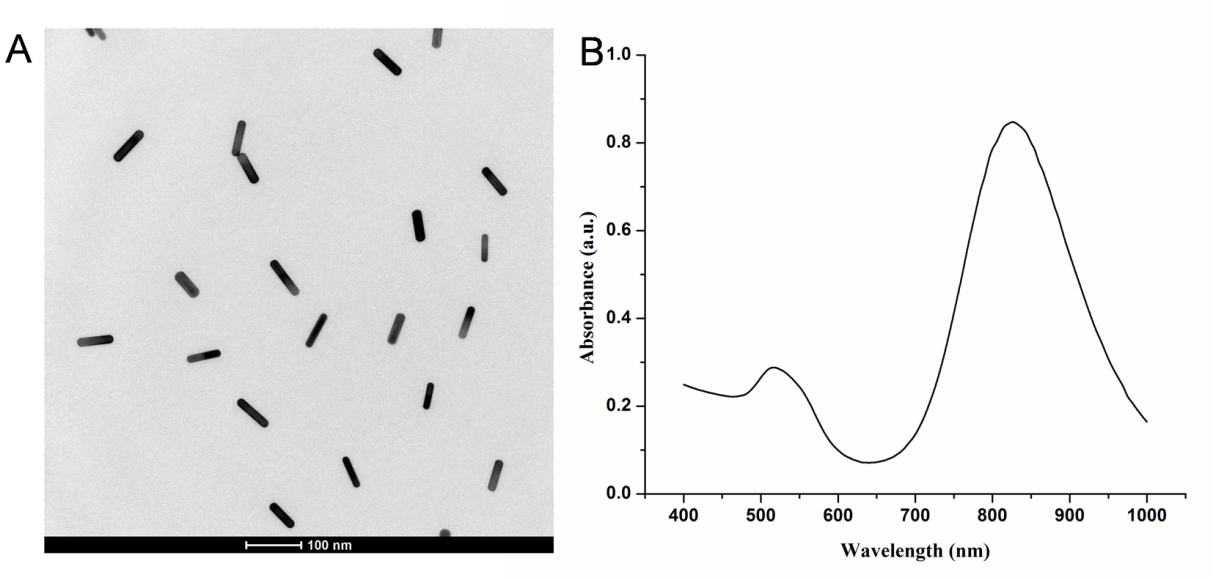


**Figure S3.** (**A**) TEM image of GNRs. Scale bar, 100 nm; (**B**) absorption spectra of GNRs.


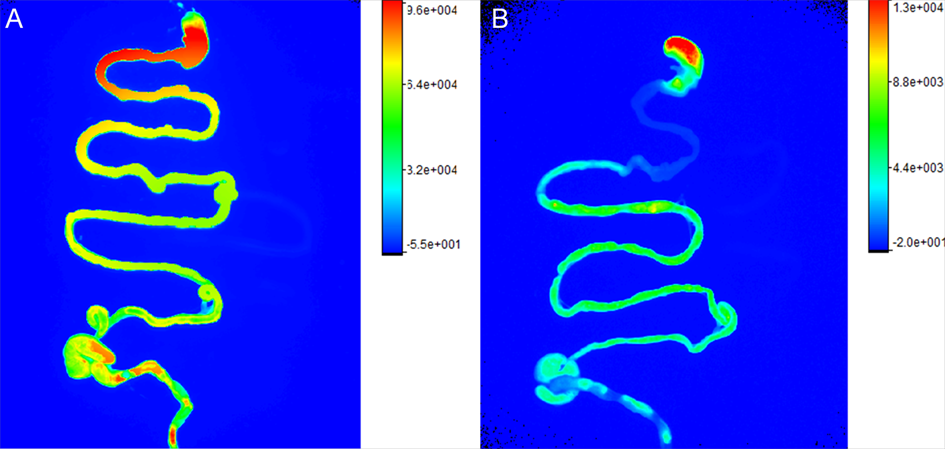


**Figure S4.** Distribution of Au nanoclusters in intestinal tissues. (A) Control groups; (B) Ligation groups.


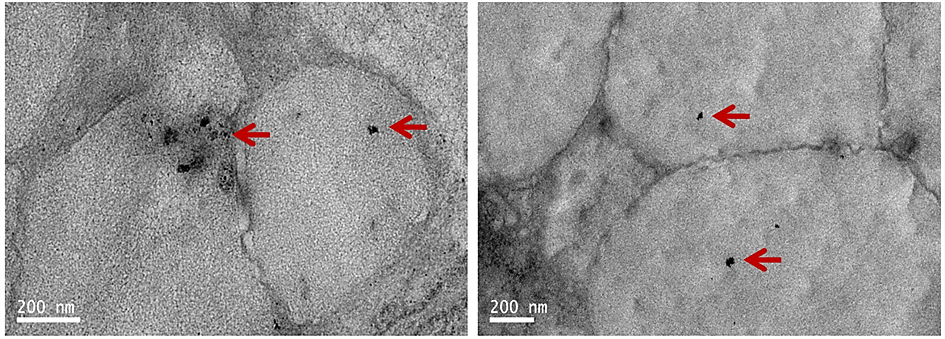


**Figure S5.** Distribution of magnetic nanoparticles in goblet cells of mice with CBD ligation.


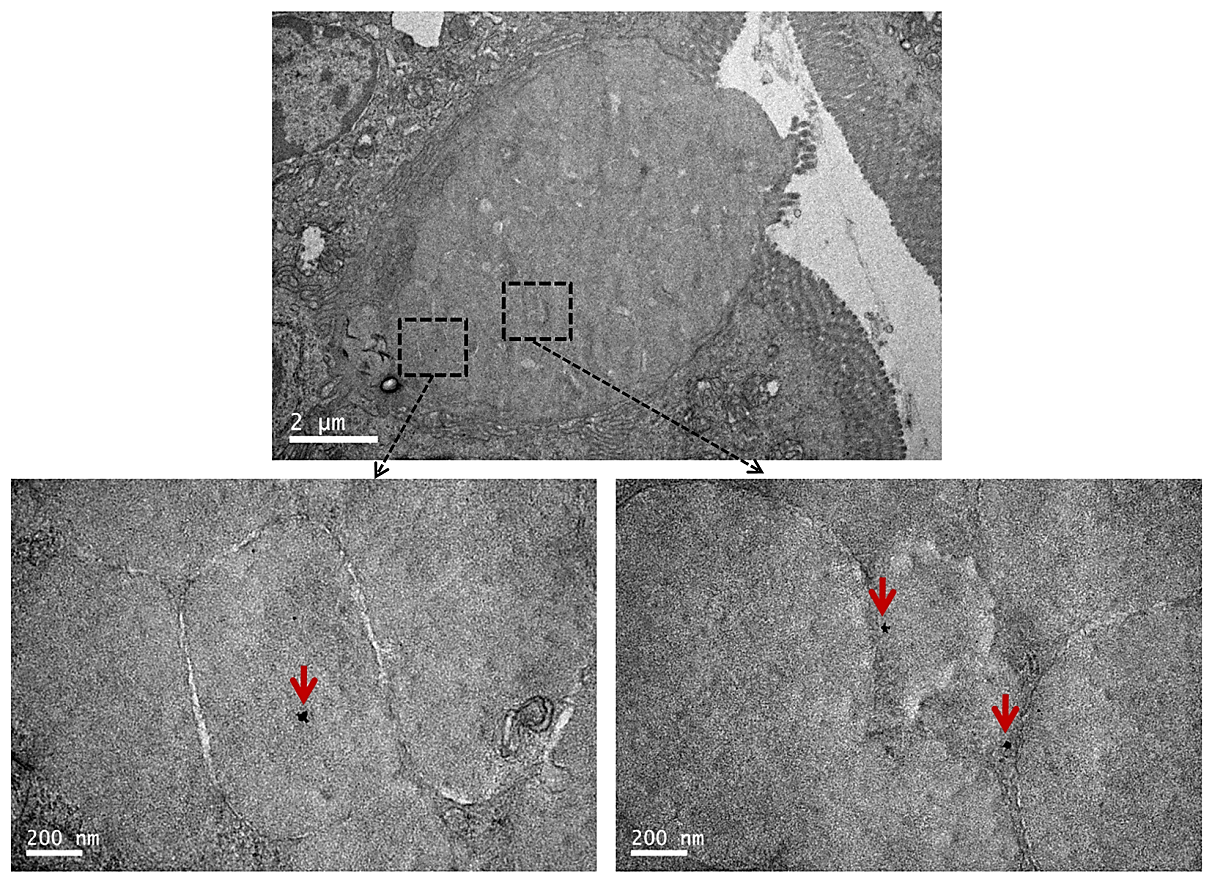


**Figure S6.** Distribution of gold nanorods in goblet cells of mice with CBD ligation.


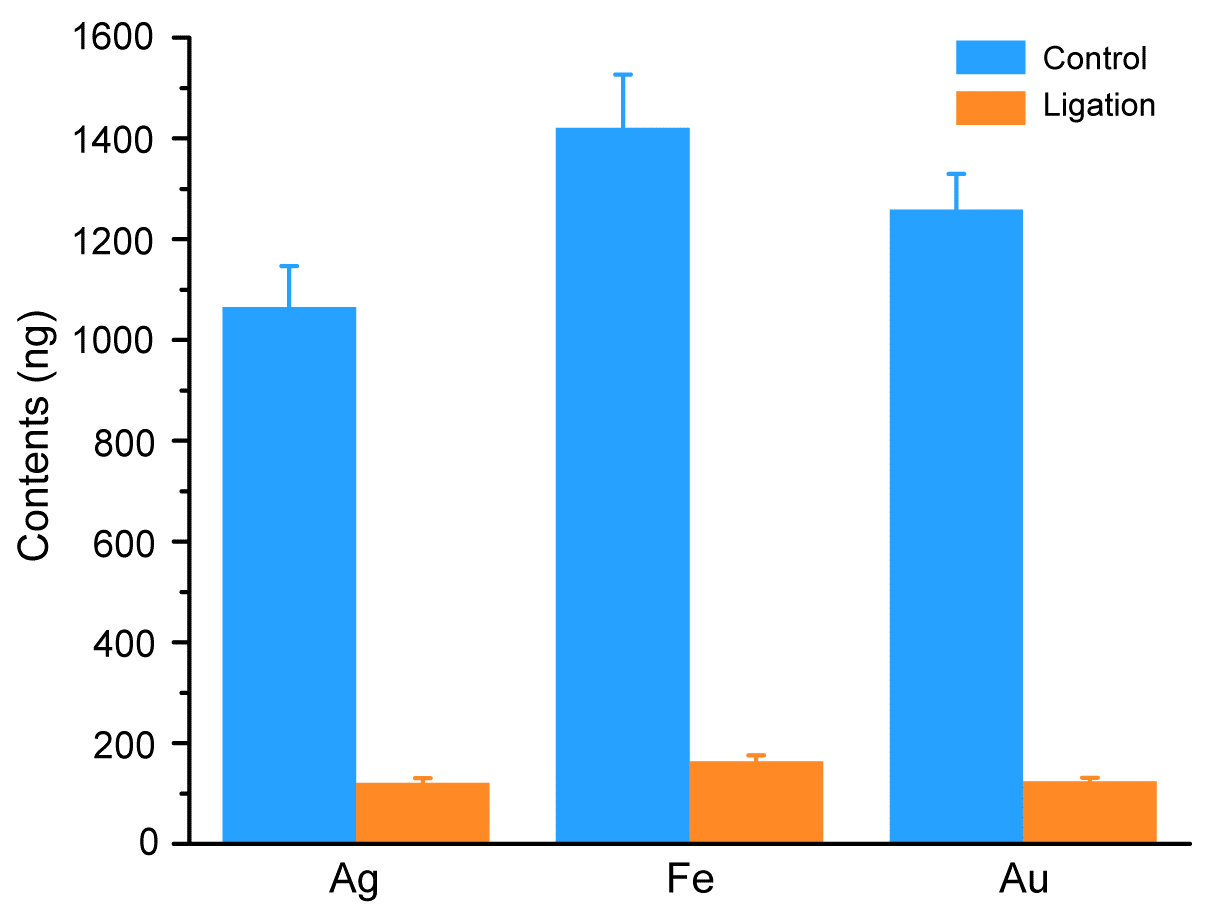


**Figure S7.** Quantitative analysis of nanoparticles in feces based on ICP-MS.
